# Utilization of early postnatal care services and associated factors among mothers who gave birth in the last 12 months in South Gondar Zone District, Amhara Regional State, Ethiopia

**DOI:** 10.1186/s41043-024-00524-4

**Published:** 2024-02-15

**Authors:** Tigist Seid Yimer, Wassie Yazie Ferede, Fillorence Ayalew Sisay

**Affiliations:** Department of Midwifery, College of Medicine and Health Sciences, Debre Tabor University, Debra Tabor, Ethiopia

**Keywords:** Factors, Early postnatal care services utilization, South Gondar Zone, Amhara region, Ethiopia

## Abstract

**Introduction:**

Postnatal care is care that is provided to mothers and newborn baby after delivery. The care given after childbirth is the most critical time because most maternal and neonatal mortality occurs during this period. Utilization of this service is low in Ethiopia, and no evidence exists to describe the status of early postnatal care service utilization among women in the study area.

**Objective:**

This study aimed to assess the utilization of early postnatal care services and associated factors among mothers who gave birth in the last 12 months in the South Gondar Zone District, Amhara Region, Ethiopia, in 2021.

**Method:**

This study was conducted in South Gondar Zone Districts from October 1 to 30, 2021. A total of 761 participants were included in this study using a simple random sampling method. The study participants were mothers who gave birth in the last 12 months. The data were collected via interview-guided semistructured questionnaires. The collected data were coded and entered into EPI Info version 7.2 and exported into SPSS version 23 for analysis. Both binary and multivariate logistic regression analyses were applied to identify factors affecting the outcome variables. The results of the final model are presented as the adjusted odds ratio (AOR) and 95% confidence interval (CI). A P value less than 0.05 was considered to indicate statistical significance.

**Results:**

In this study, 761 mothers participated, for a response rate of 100%. The overall prevalence of early utilization of postnatal care services was 20.6%. Mothers who live in urban areas were five times more likely to have early visits than those living in rural areas with adjusted odds ratio [AOR (95% CI) = 5.2 (3.19, 8.54)], a mothers who had a history of more than four parity had more likely to visit than the others at [AOR (95% CI) = 2.25 (1.18, 4.29)], mothers who had a history of pregnancy had two times more likely to visit than the other [AOR (95% CI) = 2.06 (1.05, 4.05)], and mothers who had delivered by instrumental vaginal delivery or cesarean section delivery and those mothers who had mass media exposure were two and five times more likely to visit, respectively [AOR (95% CI) = 2.62 (1.40, 4.91)] and [AOR (95% CI) = 5.18 (2.55, 10.52)].

**Conclusion and recommendation:**

Compared with those of other studies, the overall prevalence of early utilization of postnatal care services was low. Improving mothers’ knowledge of early postnatal care visits is very important for enhancing quality of life and minimizing neonatal and maternal morbidity and mortality.

## Introduction

The postnatal period was defined as the period following childbirth of a few weeks. The WHO recommends a total of 4 visits: the first visit on day. In the first 24 h, the second visit occurs on the third day, and the third visit occurs between 7 and 14, fourth visit 6 weeks after childbirth. The purpose of the visit is to identify any maternal or neonatal complications and provide appropriate care for both women and newborns [1, 2]. This period is the most critical time because most maternal and neonatal mortalities occur, and more than 50% of postnatal maternal deaths occur within the first 24 h [2]. More than 66% of deaths occur within the first week [3]. Globally, in 2017, approximately 295,000 women worldwide lost their lives due to complications occurring during pregnancy, childbirth, or the postpartum period every year, 94% of which were contributed by low-income countries. Sub-Saharan Africa and southern Asia accounted for approximately 86% (254,000) of the estimated global maternal deaths. Sub-Saharan Africa alone accounts for almost two-thirds or 66% (196,000) of those deaths [4]. Ethiopia has one of the highest maternal mortality rates (MMRs) worldwide, with 412 maternal deaths per 100,000 live births. The incidence of these deaths decreases with increasing time from birth [5, 6]. The majority of maternal and infant mortalities occur in the first month after birth. Almost half of postnatal maternal deaths occur within the first 24 h, 66% occur during the first week after delivery, and one million newborns die on the first day of life. The main reasons for these easily preventable problems were poor quality of service, weak community-based health practices, gender inequality, and poor women-centered maternity care [7, 8].

The WHO recommends four postnatal care visits [9]. The Ethiopian Federal Ministry of Health recommends four postnatal care visits at 6–24 h, 3 days, 6 days, and 42 days. The first 24 h after birth are the most critical time points for diagnosing complications and providing suitable interventions [10, 11]. Early postnatal care offers an opportunity for women to discuss healthy behaviors with providers, such as exclusive breastfeeding, proper nutrition during breastfeeding, and effective family planning [12]. In many developing countries, early postnatal care is still used at very low levels [13–16]. In Ethiopia, the impact of low coverage of early postnatal care is reflected in high maternal mortality [5, 17]. To achieve the SDGs, Ethiopia had a plan to decrease the MMR from 420 to 199/100,000 and the neonatal mortality rate from 29 to 10/1000 live births between 2015 and 2016–12019/20. The provision of early postnatal care is a key intervention for maternity and neonatal care services and for achieving their 2020 goals [18]. The Ethiopian government has developed a national strategy to minimize maternal and neonatal mortality and morbidity. Early postnatal care visits are used to identify and treat maternal and neonatal complications as well as to minimize morbidity and mortality. However, the early utilization of postnatal care visits is very low, and as a result, the rate of decrement in significant morbidity and mortality is too slow. Knowing the factors in the study area is important for informing the community, health care providers, and other concerned bodies about appropriate evidence-based reasons [19].

There are limited studies on the early utilization of postnatal care services and related factors in Ethiopia. Particularly in the Amhara region, no study has been performed. This study aimed to assess the early utilization of postnatal care services and the associated factors. These factors are crucial for decision makers (Ethiopian Federal Ministry of Health, Regional Health Bureaus, WHO, CDC, and other civil society organizations) at different levels for designing empirical and evidence-based interventions at the community level and for a policy review to comply with national and global goals.

## Methods and materials

### Study area

The study was conducted in the South Gondar Zone in three districts (Tach Gay int Woreda, Fogera Woreda, and Ebinat Woreda). South Gondar is a zone in the Ethiopian Amhara Region. The South Gondar Zone is bordered to the south by East Gojam, to the southwest by West Gojam and Bahir Dar, to the west by Lake Tana, to the north by North Gondar, to the northeast by Wag Hemra, to the east by North Wollo, and to the southeast by South Wollo. A total of 468,238 households were counted in this zone, which resulted in an average of 4.38 persons per household. There are 18 districts (10 rural and eight urban) in this zone. There are 96 health centers, 7 primary hospitals, and 1 general hospital in the zone. According to the 2011 CSA, the South Gondar Zone has a total population of 2,239,077 (female 1,103,490 males 1,135,587).

#### Study design and period

A community-based cross-sectional study was conducted from October 1 to 30, 2021.

### Source and study population

Mothers who gave birth in South Gondar Zone Districts were the source population. The mothers who gave birth in the past 12 months in the selected district were the study population.

### Inclusion and exclusion criteria

Mothers who resided for at least six months in the South Gondar Zone in the selected districts and who gave birth within the last 12 months were included. Mothers unable to hear and speak and who had psychiatric problems were excluded.

### Sample size determination

The sample size was determined by considering the single population proportion formula by assuming the prevalence of early postnatal care visit utilization (34%), a confidence level of 95%, a margin of error of 5, and a design effect of 2. Therefore, the calculated sample size of the prevalence of early utilization of postnatal care visits is as follows.

The prevalence of early utilization of postnatal care visits among women in a previous study was 34% (*p* = 0.34) at Adigrat [20].

$$n = \left( {Za/2} \right)^{2} * \, P \, \left( {1 - P} \right)/d^{{2*{\text{ design}}\;{\text{effect}}}}$$

$$n = \left( {{1}.{96}} \right)^{{2}} *0.{343 }\left( {{1}.0 - 0.{343}} \right)/0.0{5}^{{{2 }*{\text{ design}}\;{\text{effect}}\;({2})}} = { 692}$$

After considering a 10% nonresponse rate and a design effect of 2 added to the sample size calculated above, the total sample size was determined.

$$nf = {\text{final}}\;{\text{sample}}\;{\text{size}} = {761}$$

### Sampling method and procedure

**Fig. 1:**
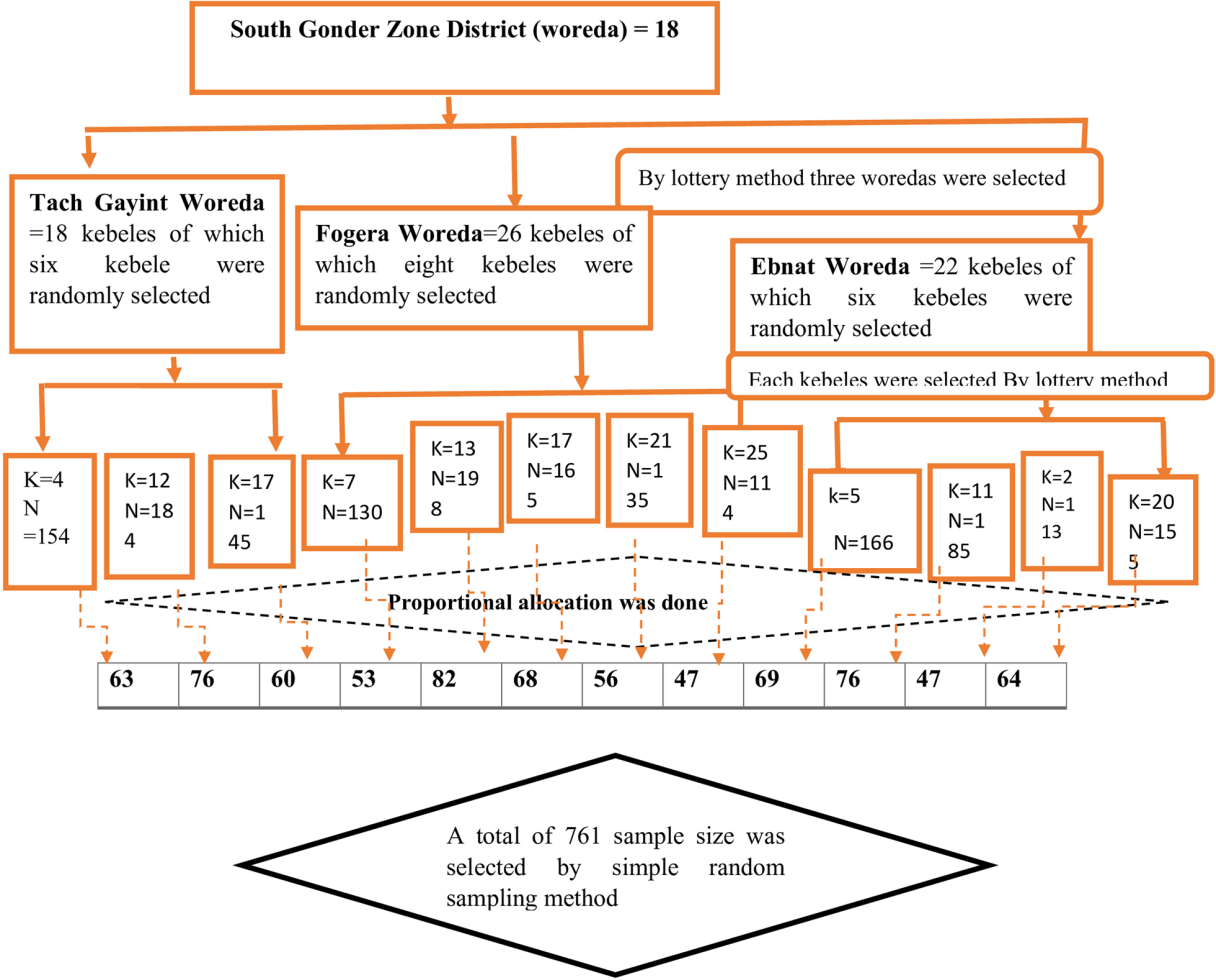
Schematic presentation of sampling procedures for the early utilization of postpartum visits in South Gondar Zone Districts, Amhara Region, Ethiopia, 2021

A total of 18 districts or woredas are found in the South Gondar Zone, three of which were selected by lottery methods. A total of 12 kebeles were selected by lottery methods. Based on the number of mothers who gave birth in the last 12 months in each kebeles from the data taken from the health extension workers, proportional allocation was performed for each kebele. Finally, the data were collected by a simple random sampling method. See Fig. 1*.*

### Variables

#### Dependent variables

Early Utilization of Postnatal Care Services (yes/no).

#### Independent variables

##### Sociodemographic factors

Age, marital status, residency status, educational status of the mothers, housebound income, and utilization of mass media.

##### Obstetric complications and reproductive characteristics

Parity, history of PNC utilization, mode of delivery, number of live births, history of abortion, current pregnancy status, number of ANC visits, any complication during pregnancy, such as antepartum hemorrhage, premature rupture of membrane, labor, and delivery, and after delivery.

##### Healthcare providers and facility-related factors

Distance of health facility, place of delivery, appointment by health care providers for early PNC, length of stay at a health facility after delivery

##### Awareness and knowledge of the client about the early utilization of postnatal care services

Awareness about the benefit of early PNC, awareness about danger signs of the mother and the newborn, and attitudes about early PNC

### Operational definition

*Early utilization of postnatal care services*: Mother at least one postnatal care checkup within 2 to 7 days [20].

*Knowledgeable*: Knowledge of the mothers about postnatal maternal danger signs, newborn danger signs, and early postnatal services provided was measured by 15 items. Mothers who scored above the mean value were considered knowledgeable.

*Not knowledgeable*: Scores below the mean value were considered.

*Satisfactory attitude*: 8 questions were applied or used to assess the attitudes of the respondents. Respondents who had an answer score above the mean value were considered to have a satisfactory attitude.

*Unsatisfactory attitude*: Patients with scores less than the mean value were considered.

### Data collection tool and procedure

The data were collected via interview-guided semistructured questionnaires. The questionnaire was developed from different studies [17, 21]. The English version was prepared and translated to the local language (Amharic); then, it was retranslated again to English by language experts to ensure consistency. The questionnaire components included demographic information, obstetric complications, and reproductive characteristic assessment questions about early PNC visit benefits, health care providers, and facility-related factors. The data collectors were 5 BSC nurses and 5 health extension workers at each site. Two supervisors (public health experts) were assigned to supervise the data collectors.

### Data quality assurance

To maintain data quality, a structured questionnaire was prepared after an extensive search and review of relevant studies on the issue. The questionnaire was subsequently translated into the local language (Amharic) to facilitate communication. The supervisor and data collectors were trained for two days on how to collect the data, study overviews, and questionnaires and how to perform other related activities during the data collection.

In addition, the structured questionnaire was pretested on 5% of the total sample size outside the study area. Difficult questions were revised, and modifications were carried out after the pretest. Daily supervision of the data collection process was maintained throughout the data collection period. Supervisors checked the study sites daily and received completed questionnaires after checking for completeness.

### Data processing and analysis

Before data processing, the data were checked for completeness and internal consistency. The data were coded and entered into EPI Info version 7.2 and exported to SPSS version 23 for analysis. The analyses were verified using descriptive interpretation of the socioeconomic characteristics of the study participants using frequencies and other summary statistics. Binary logistic regression was used to measure the association of each covariate with the outcome variable. Factors that were associated with the outcome variable at the 20% significance level were included in the multivariable logistic regression analysis to control for potential confounders. The results of the final model were presented as the adjusted odds ratio (AOR) and 95% confidence interval (CI), and a P value less than 0.05 was considered to indicate statistical significance.

## Results

### Sociodemographic characteristics

In this study, the majority of the participants were aged 21 to 25 years. of 281 (37.2%), married 587 (77.6%), Amhara in Ethnicity 676 (89.4%), Orthodox followers 688 (91.0%), and rural residents 584 (77.2%).

Regarding maternal and husband educational status, the majority of the mothers were able to read and write (36.6%). Forty-seven percent of the respondents’ income levels were within the range of 1000 to 1500 ETB. The majority of the respondents (83.3%) did not utilize mass media. Regarding distances from the health institution and place of delivery, the majority of the respondents had traveled more than 1 h on foot to reach the health facility. Eighty percent of the respondents were delivered to governmental health institutions. More than 70.8% of the mothers said that they did not have an appointment with the health care providers when they returned to the hospital for early postnatal care visits. A total of 69.7% of the mothers stayed at the health facility for less than 24 h (Table 1)*.*

**Table 1:** Sociodemographic and socioeconomic characteristics of the respondents at South Gondar Zone District, Amhara Region, Ethiopia, 2022 (*n* = 761)

Variable	Characteristics	Number	Percentage
Age	< 20 yrs	45	6.0
	21 to 25 yrs	281	37.2
	26 to 30 yrs	174	23
	31 to 35 yrs	112	14.8
	36 to 40 yrs	144	19.0
Marital status	Married	587	77.6
	Unmarried	169	22.4
Religion	Orthodox	688	91.0
	Muslim	68	9.0
Ethnicity	Amhara	676	89.4
	Tigire	80	10.6
Resident	Rural	584	77.2
	Urban	172	22.8
Mother educational status	Unable to read and write	277	36.6
	Able to read and write	302	39.9
	Primary school	62	8.2
	Secondary school	40	5.3
	College and above	75	9.9
Husband educational status	Unable to read and write	170	22.5
	Able to read and write	159	21.0
	Primary	312	41.3
	Secondary	23	3.0
	College and above	92	12.2
Income	< 1000 ETB	280	37.0
	1000 to 1500 ETB	357	47.2
	> 1500 ETB	119	15.7
Mass media utilization	No	630	83.3
	Yes	126	16.7
Distance from health facility	< 30 min	112	14.8
	30 min to 1 h	124	16.4
	> 1 h	520	68.8
Place of delivery	Home delivery	96	12.7
	Governmental health institution	603	79.8
	Private health institution	57	7.5
Had an appointment with the health care providers when to return	No	535	70.8
	Yes	221	29.2
Length of stay at a health facility	< 24 h	527	69.7
	> 24	133	17.6

#### Reproductive characteristics and obstetric complications

More than 60% of the respondents had para four or above. Only 26.7% of the mothers had postnatal care visits, and 20.6% of the respondents had early postnatal care utilization. A total of 23.7% of the mothers had two visits. Regarding the place of visit, 26.2% were at health centers.

Eighty-six percent of the respondents had a history of previous PNC utilization. Regarding the mode of delivery, 88% of the respondents delivered via spontaneous vaginal delivery. Of the respondents, 83.9% had a history of ANC care utilization. More than 65.5%, 95% and 90% of the mothers had no complications during pregnancy, during labor and delivery, or after delivery, respectively (Table 2).

**Table 2:** reproductive characteristics and Obstetric complication at South Gondar Zone District, Amhara Region, Ethiopia, 2021 (*n* = 761)

Variable	Characteristics	Numbers	Percentage (%)
Parity	< 4	237	31.3
	≥ 4	519	68.7
PNC visit	No	554	73.3
	Yes	202	26.7
Time of visit	24 h to 3 days	156	20.6
	4 to 7 days	42	5.6
	8 to 42 days	4	0.5
Frequency of visit	One time	12	1.6
	Two times	179	23.7
	Three and above	17	2.2
Place of visit	No visit	554	73.3
	Home	4	0.5
	Health center	198	26.2
History of PNC use	No	136	18.0
	Yes	620	82.0
Mode of delivery	SVD	666	88.1
	Instrumental vaginal delivery and c/s	90	11.9
Number of live birth	No	84	11.1
	Yes	672	88.9
History of abortion	No	551	72.9
	Yes	205	27.1
ANC use	No	122	16.1
	Yes	634	83.9
condition of px	Unplanned unwanted and unsupported	282	37.3
	Planned, wanted, and supported	474	62.7
Complication during pregnancy	No	493	65.2
	Yes	263	34.8
Complications during labor and delivery	No	722	95.5
	Yes	34	4.5
Complications after labor and delivery	No	684	90.5
	Yes	72	9.5

#### Knowledge and attitude of the respondent toward early PNC visits

Regarding the knowledge and attitude of the respondents toward early PNC visits, 70% of the respondents had no knowledge, and only 27% of the mothers had a satisfactory attitude (Table 3)*.*

**Table 3:** Knowledge and attitude of the respondent toward early PNC visit at South Gondar Zone District, Amhara Region, Ethiopia, 2021 (n = 761)

Variable	Characteristics	Numbers	Percentage
Knowledge about early PNC visit	No	550	72.8
	Yes	206	27.2
The attitude of the respondent toward early PNC visit	Unsatisfactory attitude	552	73.0
	Satisfactory attitude	204	27.0

#### Factors associated with early utilization of PNC

According to our bivariate analysis, marital status, residency, parity, history of pregnancy, mode of delivery, number of live births, and complications after delivery were associated with early postnatal care utilization. Both bivariate and multivariate analyses showed that residence, history of pregnancy, mode of delivery, parity, and mass media exposure were significantly associated with early postnatal care utilization. Mothers who live in urban areas were five times more likely to have early utilization than those living in rural areas [AOR (95% CI) = 5.2 (3.19, 8.54)], parity mothers who had a history of more than four parity had more likely to visit than the others at [AOR (95% CI) = 2.25 (1.18, 4.29)], mothers who had a history of pregnancy had two times more likely to visit than the other [AOR (95% CI) = 2.06 (1.05, 4.05)], and mothers who had delivered by instrumental vaginal delivery and those mothers who had a history of mass media exposure were two and five times more likely for early PNC utilization [AOR (95% CI) = 2.62 (1.40, 4.91)] and [AOR (95% CI) = 5.18 (2.55, 10.52)], respectively (Table 4)*.*

**Table 4:** Factors associated with utilization of early postnatal care services at South Gondar Zone District, Amhara Region, Ethiopia, 2021 (*n* = 761)

Variable	Category			OR (95% CI)	A0R (95% CI)
		Had no early utilization of postnatal care	Had early utilization of postnatal care		
Resident	Rural	521	63		1
	Urban	79	93	9.73 (6.54, 14.49)*	5.2 (3.19, 8.54)**
Parity	≤ 4	219	18	1	1
	> 4	381	138	4.40 (2.62, 7.40)*	2.25 (1.18, 4.29)**
History of pregnancy	No	122	14	1	1
	Yes	478	142	2.58 (1.44, 4.64)*	2.06 (1.05, 4.05)**
Mode of delivery	SVD	545	121	1	1
	Instrumental	55	35	2.86 (1.79, 4.57)*	2.62 (1.40, 4.91)**
Number of live births	No	82	2		1
	Yes	518	154	0.082 (.020,.337)*	1.22 (0.276, 5.438)
Mass media exposure	No	579	105	1	1
	Yes	21	51	13.3 (27.73, 23.19)*	5.18 (2.55, 10.52)**

* = significant at **p**-value <0.05 and ** = significant at **p**-value <0.001

## Discussion

This study included only 20.6% (95% CI 17.6–23.4) of the women’s early postnatal care visits. These findings are lower than those of studies conducted in Adigrat town, Tigray, 34.3% [21]; Yirgalem town, Sidma, Ethiopia, 45.5% [22]; Hawassa Zuria District 27.7% [23]; and Lemmo District, Hadiya Zone, 24.9% [17]. This variation might be due to the difference in the time lapse between the studies. The study was performed in Hawassa Zuria District with mothers who gave birth in the last six months; however, in this study, the study was performed with mothers who gave birth in the last twelve months.

On the other hand, these findings are similar to those of cross-sectional studies conducted in the Sidama Zone Malga district (22.5%) [24], Wolkite town, Gurage Zone (22.3%) [25], Aseko District, Arsi Zone (23.7%) [26], and Rural Women in the Horo Guduru Wollega Zone (21.8%) [27]. This might be due to similarities in the sociodemographic characteristics of the respondents. Like in the Sidama Zone Malga district, the majority of the women were married (98.5%).

In addition, these findings are lower than those of other studies conducted in Uganda (65%) [28], Eastern Uganda (55%) [29], Myanmar (72.1%) [30], and Zambia (63%) [31]. This difference might be due to the difference in approaches to implementing early PNC service provision differences in socioeconomic status, geographical barriers, accessibility of services, and health education adopted in early PNC service provision between countries.

In addition, more of these findings are consistent with those of a study conducted in Wonago District, southern Ethiopia, in which 13% of the participants were affected [32]. The variation in the study might be due to the difference in the time lapse. The study was conducted in Wonago District with mothers who gave birth in the last six months. This study was conducted on mothers who gave birth in the last twelve months. Another possible explanation might be the difference in the provision of early postnatal care services.

On the other hand, the result of this finding is higher than the cross-sectional study conducted in Mundri East County, South Sudan (11.4%) [33]. A possible explanation might be differences in the quality of health care and the implementation of service provision.

In this study, mothers who lived in urban areas were five times more likely to have early PNC visits than were those living in rural areas [AOR (95% CI) = 5.2 (3.19, 8.54)]. This result is supported by a study performed in Uganda’s 2016 Demographic and Health Survey [34, 35]. A possible explanation might be that rural areas have less access to public services, such as roads, transport, and health services. As a result, urban residents are likely to have more access to transportation and healthcare services, which results in increased utilization of early postnatal services.

The odds of getting an early PNC visit were 2.25 times higher among mothers who had a history of more than four parities than among those who did not [AOR (95% CI) = 2.25 (1.18, 4.29)]. Gurage Zone [25], Lemmo District, Hadiya Zone [17]. This might be due to previous pregnancies sharing the experience of attending early postnatal visits, and the number of births increases the mothers seeking obstetric care and utilizing early postnatal services.

This study revealed that mothers who had a history of pregnancy were two times more likely to have early PNC visits than mothers with no history of pregnancy [AOR (95% CI) = 2.06 (1.05, 4.05)], which might be due to the history of pregnancy, the knowledge provided during antenatal care, and the importance of postnatal care at childbirth having a positive influence on the postnatal health-seeking behavior of women who access these services.

This study revealed that women who gave birth by instrumental vaginal delivery or cesarean section delivery were 2.6 times more likely to have early PNC visits than women who gave birth by spontaneous vaginal delivery [AOR (95% CI) = 2.62 (1.40, 4.91)]. These findings are similar to those of other studies performed in the Sidama Regional State, Ethiopia [22], in the Lemmo District, Hadiya Zone [17], and Uganda health facilities [28]. A possible explanation might be that women who have instrumental delivery are more likely to suffer from physical pain after childbirth and have longer and more difficult postnatal recovery, both conditions that also affect their psychological well-being because they may increase early PNC utilization.

Compared with mothers who had no mass media exposure, respondents who had a history of mass media exposure had increased early utilization of early PNC five times [AOR (95% CI) = 5.18 (2.55, 10.52)].

## Conclusion and recommendation

In general, this study revealed that the coverage of early postnatal care service utilization was lower in the study area compared with other studies. Urban residents of the respondents, having areas. A history of pregnancy, instrumental delivery or cesarean section delivery, a history of mass media exposure, and a parity greater than four were factors that were significantly associated with EPNC service usage. The overall prevalence of early utilization of postnatal care services was lower than that reported in other studies. Improving mothers’ knowledge of early postnatal care visits is very important for enhancing quality of life and minimizing neonatal and maternal morbidity and mortality.

## Data Availability

However, the data sets collected and analyzed for the current study are available from the corresponding author and can be obtained upon reasonable request.

## Abbreviations

ANC: Antenatal care
AOR: Adjusted odds ratio
CI: Confidence interval
COR: Crude odds ratio
EPNC: Early postnatal care
ETB: Ethiopian border
PNC: Postnatal care
SPSS: Statistical Package for Social Science
WHO: World Health Organization
